# Supplementary material for: In-depth correlation analysis between tear glucose and blood glucose using a wireless smart contact lens
Source: Nat Commun. 2024 Apr 2;15:2828. doi: 10.1038/s41467-024-47123-9 (PMC10987615; doi:10.1038/s41467-024-47123-9)
Supplement: Supplementary file 1 — Supplementary Information [file 41467_2024_47123_MOESM1_ESM.pdf]

In-depth correlation analysis between tear glucose and blood glucose using a wireless smart contact lens

Wonjung Park[1,2†], Hunkyu Seo[1,2†], Jeongho Kim[3†], Yeon-Mi Hong[1,2], Hayoung Song[1,2], Byung Jun Joo[1,2], Sumin Kim[1,2], Enji Kim[1,2], Che-Gyem Yae[4], Jeonghyun Kim[5], Jonghwa Jin[6], Joohee Kim[7★], Yong-ho Lee[8,9,10★], Jayoung Kim[11★], Hong Kyun Kim[3,4,12★], Jang-Ung Park[1,2,13,14★].

[1]Department of Materials Science and Engineering, Yonsei University, Seoul 03722, Republic of Korea

[2]Center for Nanomedicine, Institute for Basic Science (IBS), Yonsei University, Seoul, 03722, Republic of Korea

[3]Cell and Matrix Research Institute, School of Medicine, Kyungpook National University, Daegu, South Korea

[4]Department of Ophthalmology, Kyungpook National University School of Medicine, Daegu 41944, Republic of Korea

[5]Department of Electronics Convergence Engineering, Kwangwoon University, Seoul 01897, Republic of Korea

[6]Department of Internal Medicine, School of Medicine, Kyungpook National University, Kyungpook National University Hospital, Daegu 41944, Republic of Korea

[7]Center for Bionics, Biomedical Research Division Korea Institute of Science and Technology, Seoul 02792, Republic of Korea

[8]Department of Internal Medicine, Yonsei University College of Medicine, Seoul 03722, Republic of Korea

[9]Institute of Endocrine Research, Yonsei University College of Medicine, Seoul 03722, Republic of Korea

[10]Institute for Innovation in Digital Healthcare (IIDH), Severance Hospital, Seoul 03722, Republic of Korea

[11]Department of Medical Engineering, Yonsei University College of Medicine, Seoul 03722, Republic of Korea

[12]Department of Ophthalmology, Kyungpook National University Hospital, Daegu 41944, Republic of Korea

[13]Department of Neurosurgery, Yonsei University College of Medicine, Seoul 03722, Republic of Korea

[14]Graduate Program of Nano Biomedical Engineering (NanoBME), Advanced Science Institute, Yonsei University, Seoul 03722, Republic of Korea

★e-mail: jang-ung@yonsei.ac.kr(J.-U.P.), okeye@knu.ac.kr(H.K.K.), jayoungkim@yonsei.ac.kr(Jayoung K.), yholee@yuhs.ac(Y.L.), joohee710610@kist.re.kr(Joohee K.)

† These authors contributed equally to this work.

This supplementary information file includes:

Supplementary Figures

1. Schematic illustration on the fabrication of smart contact lens.
2. Photograph of integrated smart contact lens.
3. Cyclic voltammetry of the glucose sensor.
4. Characterization of the glucose sensor.
5. Photograph of smart contact lens on the eye of the mannequin.
6. Real-time data of glucose concentration exported from a smartphone.
7. Cell cytotoxicity test of normal contact lens and smart contact lens.
8. Fluorescence microscope images of human corneal cells and human conjunctival cells.
9. Image of tear volume measurement tool and eye stimulating tool.
10. Tear volume measurement as a function of stimulation force in each rabbit.
11. Continuous measurement of glucose level and tear volume along with corneal stimulation in normal rabbits.
12. Comparison of tear glucose level recovery time and tear volume recovery.
13. Simultaneous measurement of tear glucose concentrations in the right and left eyes of a normal rabbit.
14. Tear glucose and blood glucose level after oral administration of glucose in rabbits.
15. Calculation of Pearson's correlation coefficient and regression line between tear glucose and blood glucose for a normal rabbit.
16. Lag time identification of all measured oral glucose tolerance test in normal rabbits.
17. Comprehensive Pearson's correlation analysis of all measured oral glucose tolerance test in 4 normal rabbits.
18. Change in the average of weight and blood glucose of four rabbits after STZ treatment.
19. Comparison of pancreatic islet between normal and diabetic rabbits.

20. Real-time measurement of tear glucose and blood glucose of each normal rabbit after intravenous injection of glucose.
21. Real-time measurement of tear glucose and blood glucose of each diabetic rabbit after intravenous injection of glucose and insulin.
22. Change in the average of weight and blood glucose of four beagles after STZ treatment.
23. Comparison of pancreatic islet between normal and diabetic beagles.
24. Real-time measurement of tear glucose and blood glucose of each normal beagle after intravenous injection of glucose.
25. Real-time measurement of tear glucose and blood glucose of each diabetic beagle after intravenous injection of glucose and insulin.
26. Images of ten healthy participants wearing the SCL.
27. Images of ten diabetic participants wearing the SCL.
28. Real-time data of monitoring TG and BG level in healthy participants.
29. Real-time data of monitoring TG and BG level in diabetic participants
30. The lag time and Pearson's correlation coefficient on day 1, 3, and 5.
31. Pearson's correlation analysis between TG and BG in healthy participants.
32. Pearson's correlation analysis between TG and BG in diabetic participants.

Supplementary Figures

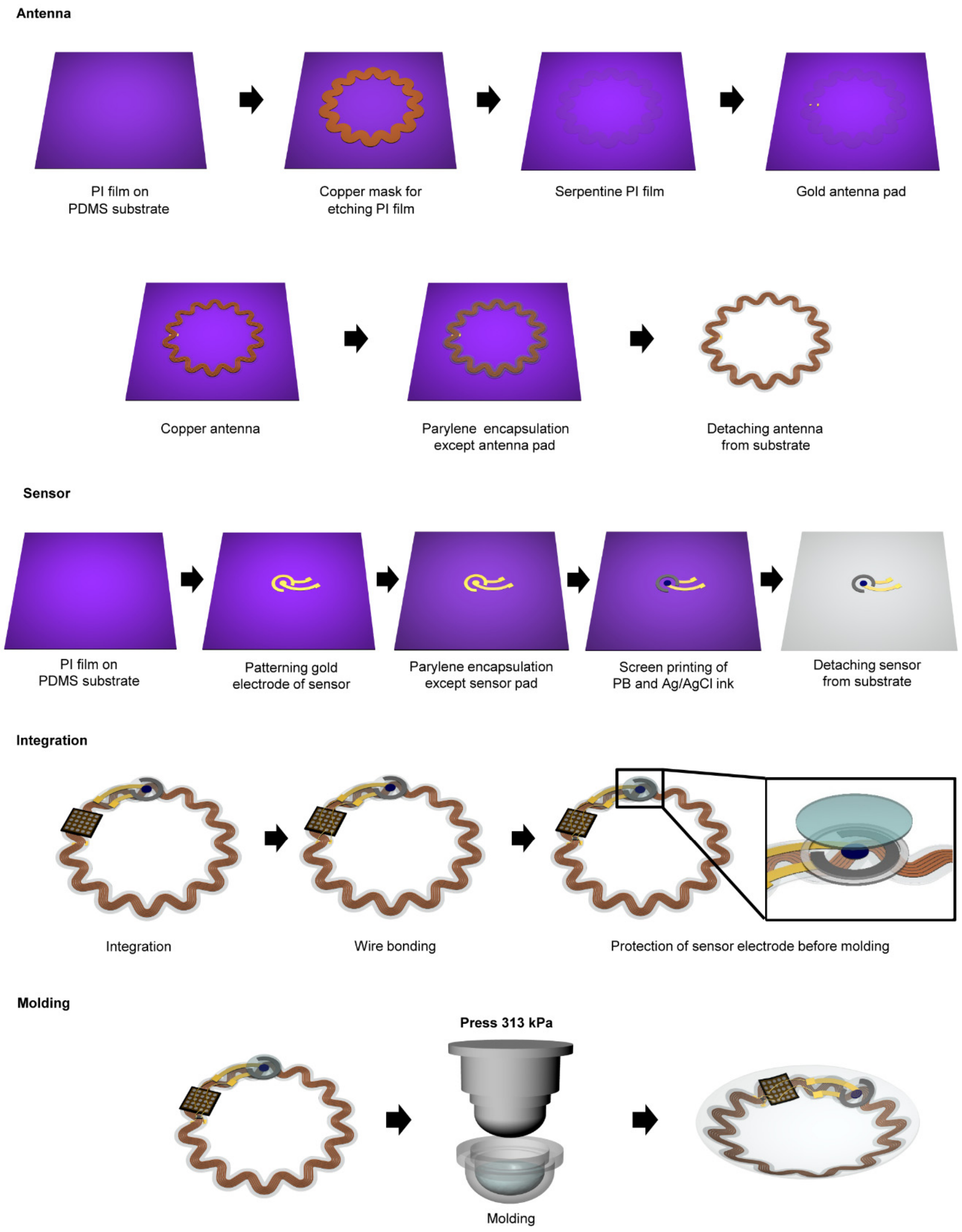

Supplementary Figure 1. Schematic illustration on the fabrication of antenna, glucose sensor, integration process, and molding process of smart contact lens.

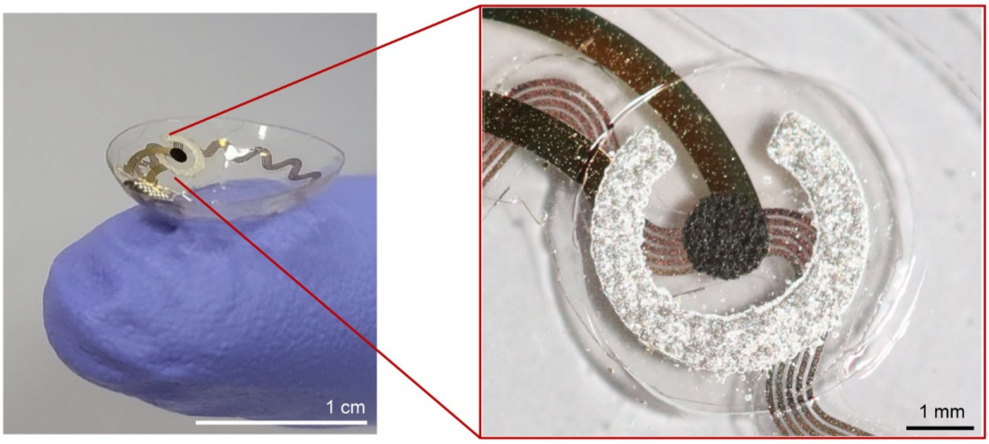

Supplementary Figure 2. Photograph of integrated smart contact lens with the exposed glucose sensor (left) and optical micrograph of the exposed glucose sensor (right).

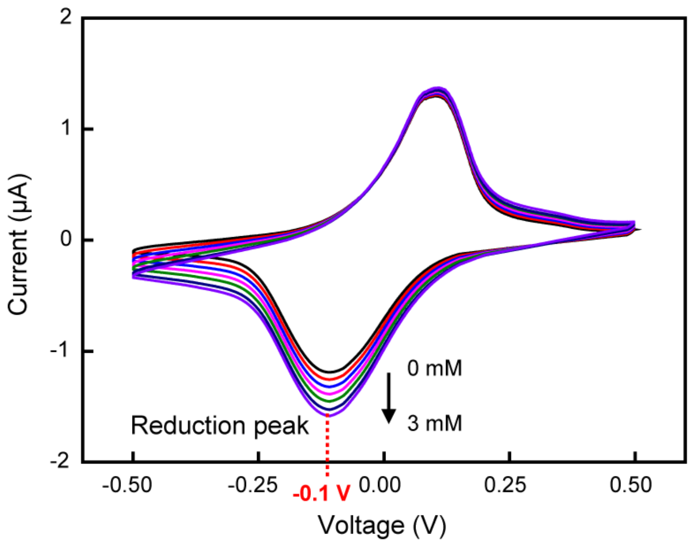

Supplementary Figure 3. Cyclic voltammetry of the glucose sensor in increasing glucose levels in the range from 0 to 3 mM with 0.5 mM increment. Scan range, −0.5 V to 0.5 V; Scan rate, 50 mV s^{-1}

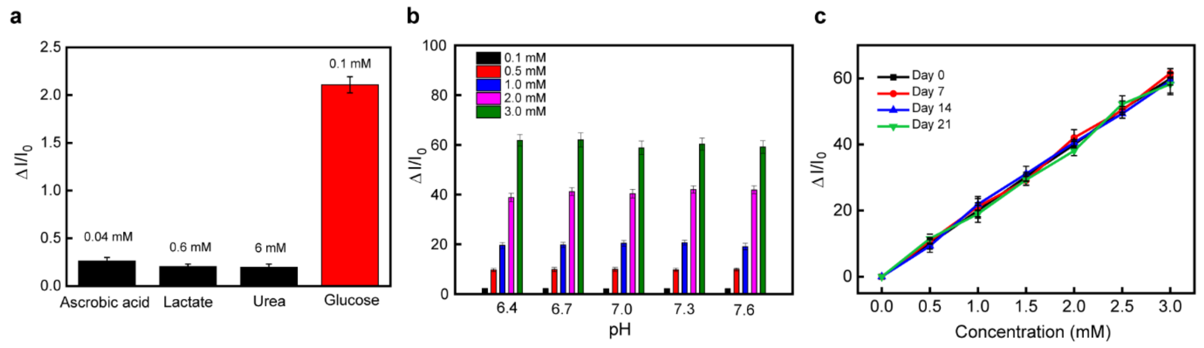

Supplementary Figure 4. Characterization of the glucose sensor. a, Relative changes of current in ascorbic acid, lactate, urea, and glucose solution. b, Relative changes in current according to the concentrations of glucose in different pH ranges from 6.4 to 7.6 with the increment of 0.3. c, Long-term stability test of the glucose sensor after being stored in PBS at room temperature for up to 21 days. For a-c, each data point represents the average of 20 samples, and the error bars indicate mean ± standard error of the mean. Each sample represents a biologically independent sample.

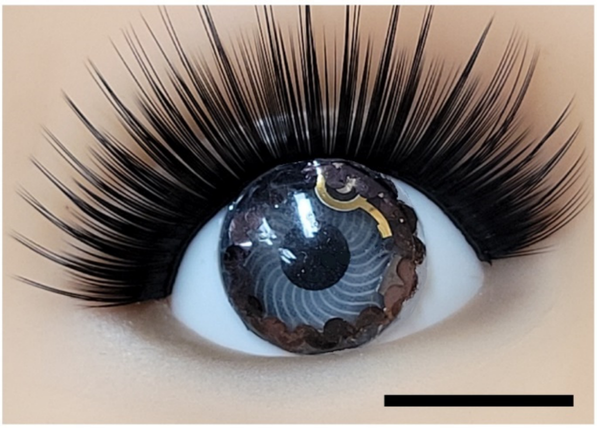

Supplementary Figure 5. Photograph of smart contact lens on the eye of the mannequin. Scale bar, 1 cm.

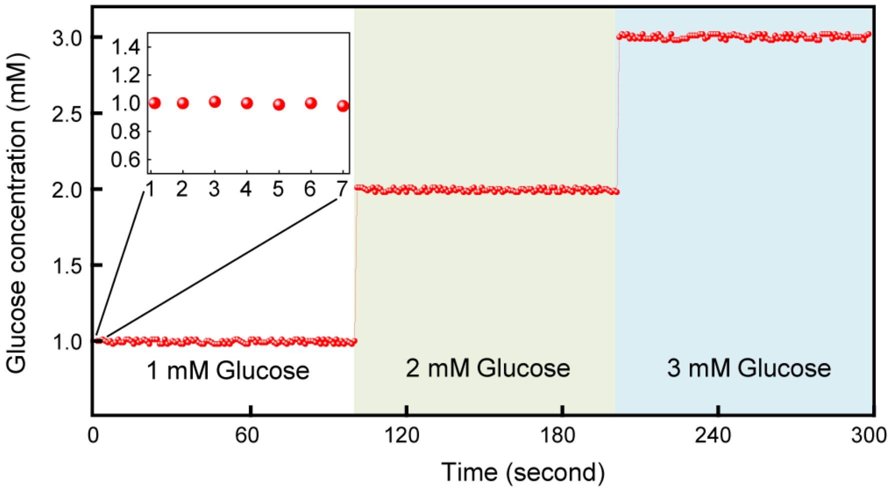

Supplementary Figure 6. Real-time data of glucose concentration exported from a smartphone after measuring glucose level using a smart contact lens worn on the mannequin eye. After flowing the PBS solution containing specific concentrations of glucose into the mannequin eyes wearing the SCL, the tagged smartphone (within a distance of 5 mm from the SCL) receives glucose information wirelessly.

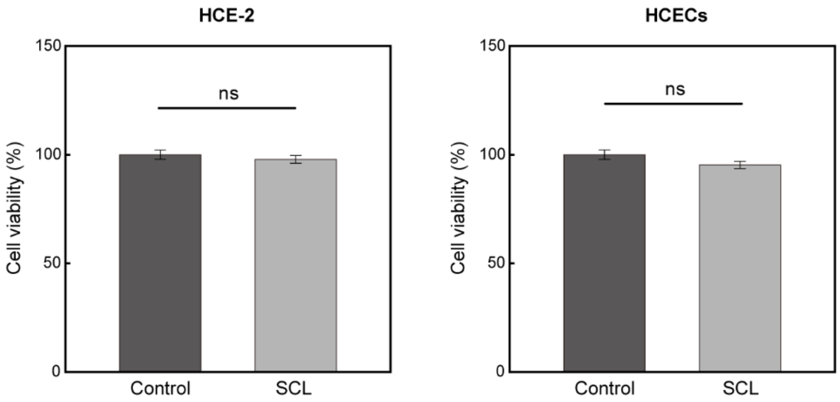

Supplementary Figure 7. Cell cytotoxicity test of normal contact lens (control) and smart contact lens (SCL) with human corneal cells (HCE-2) (n = 20) and human conjunctival cells (HCECs) (n = 20). Significant differences were analyzed with the unpaired student's t-test and marked as ns ($p > 0.05$). The error bars indicate mean ± standard error of the mean.

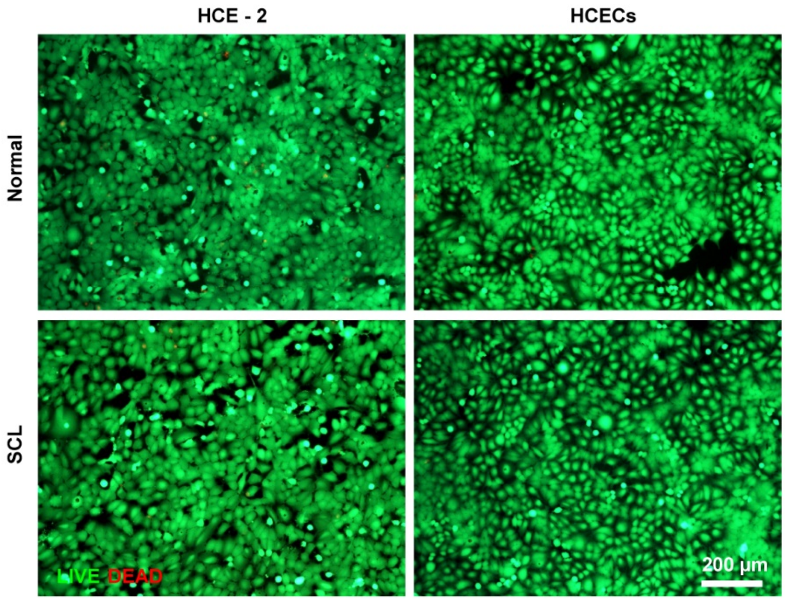

Supplementary Figure 8. Fluorescence microscope images of human corneal cells (HCE-2) and human conjunctival cells (HCECs), cultured in medium of normal contact lens (control) and smart contact lens (SCL) medium, stained with calcein-AM.

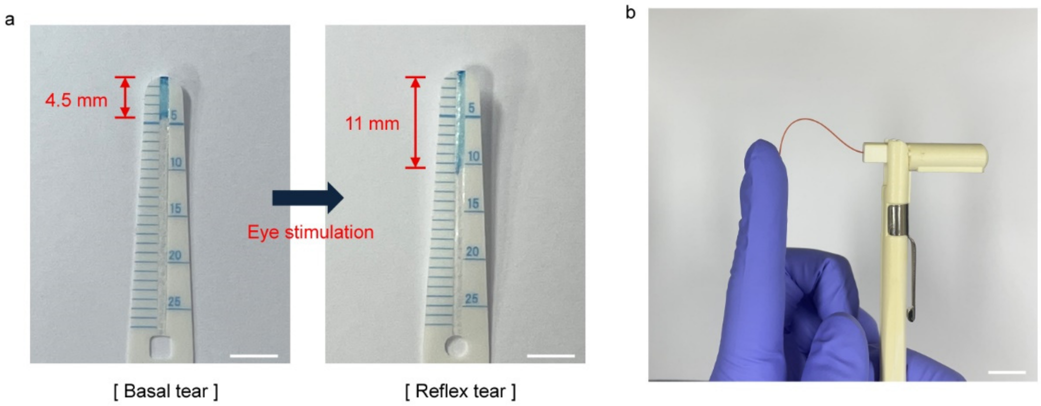

Supplementary Figure 9. a, Tear volume measurement tool (SMTube). Scale bars, 500 µm. b, Eye stimulating tool (von Frey filament). Scale bars, 1 cm.

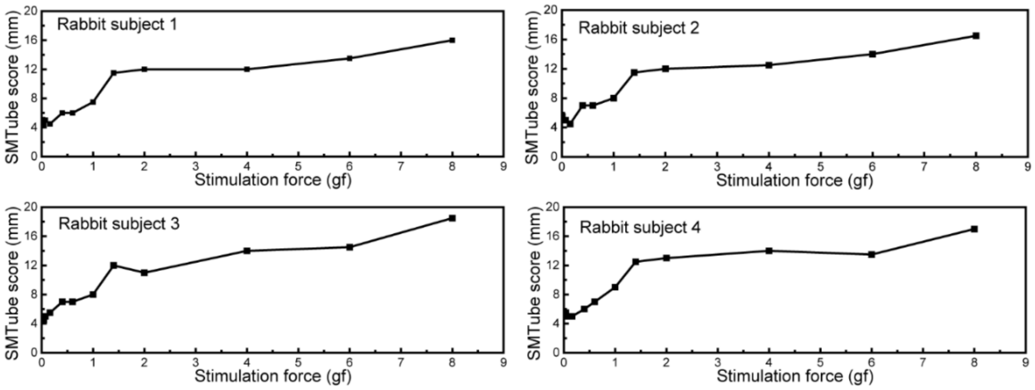

Supplementary Figure 10. Tear volume measurement as a function of stimulation force in each rabbit.

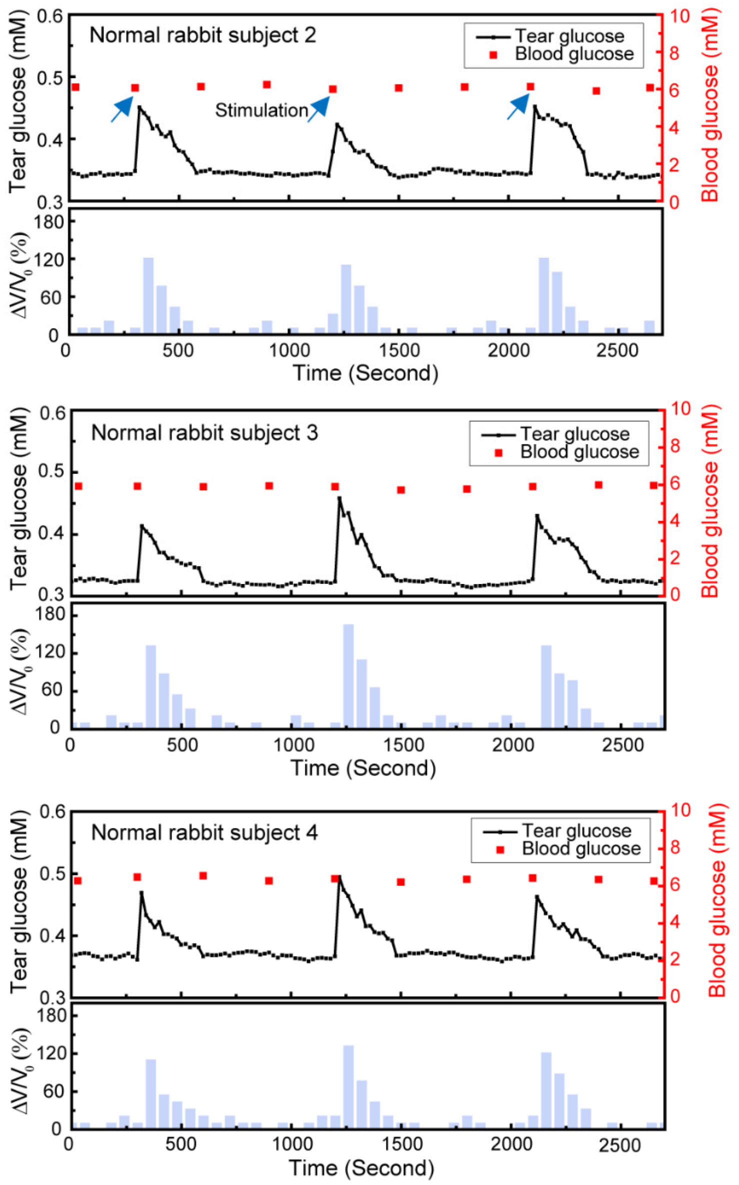

Supplementary Figure 11. Continuous measurement of glucose level and tear volume along with corneal stimulation in each normal rabbit.

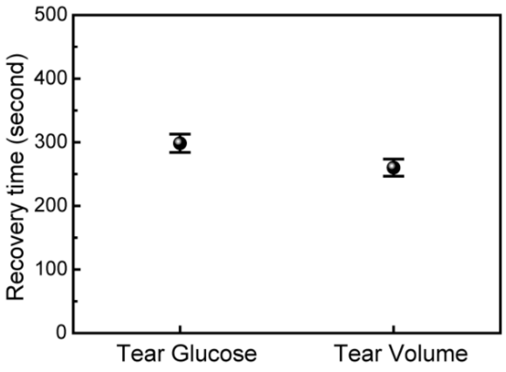

Supplementary Figure 12. Comparison of tear glucose level recovery time and tear volume recovery time of normal rabbits after mechanical stimulation (n = 4). The error bars indicate mean ± standard error of the mean.

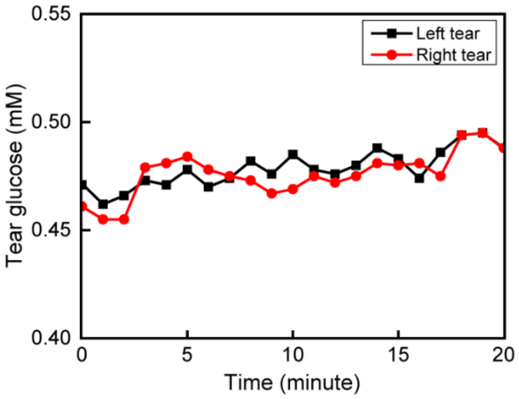

Supplementary Figure 13. Simultaneous measurement of tear glucose concentrations in the right and left eyes of a normal rabbit.

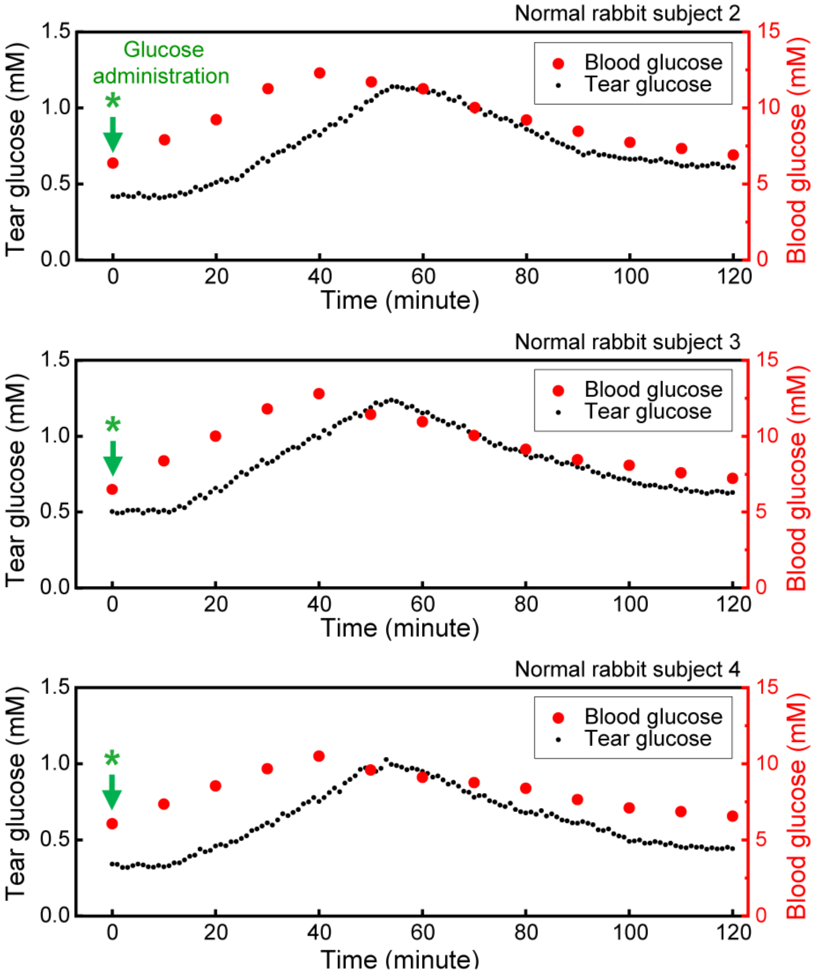

Supplementary Figure 14. Real-time measurement of tear glucose and blood glucose after oral administration of glucose in each normal rabbit.

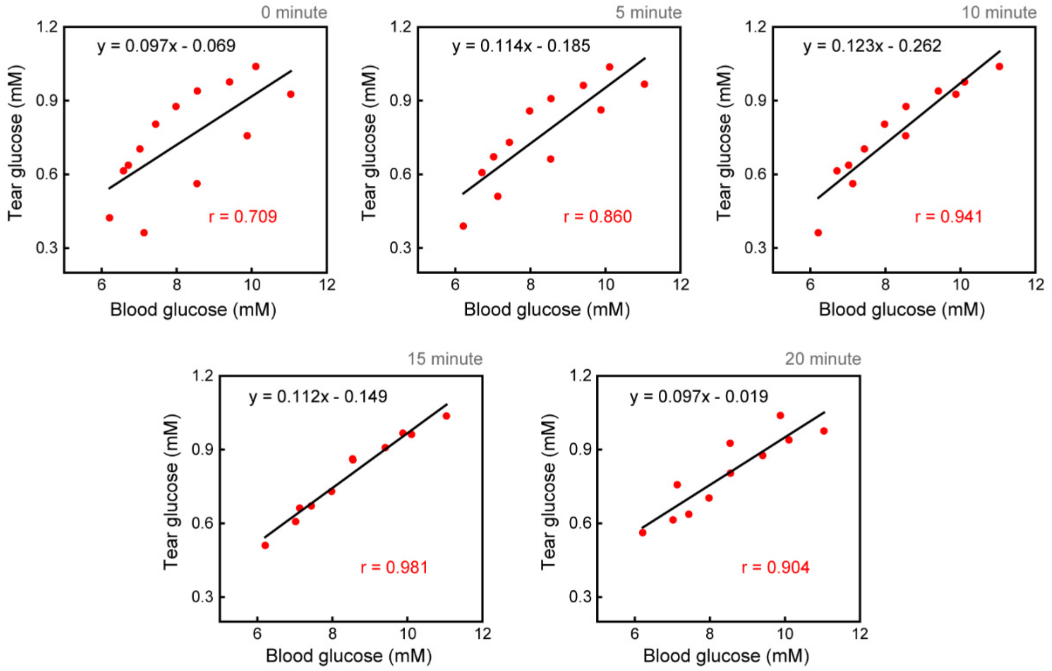

Supplementary Figure 15. Calculation of Pearson's correlation coefficient and regression line between tear glucose and blood glucose for a normal rabbit when the lag time is assumed to be 0 min, 5 min, 10 min, 15 min, and 20 min.

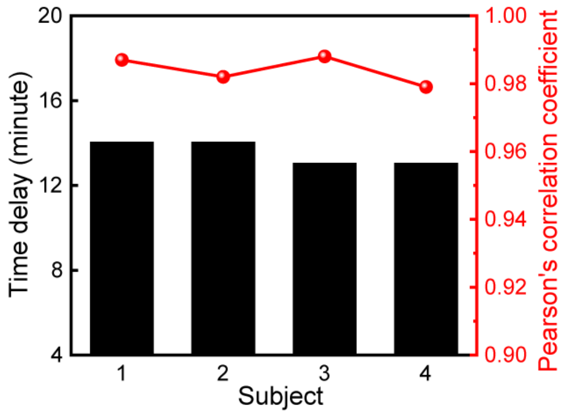

Supplementary Figure 16. Lag time identification of all measured oral glucose tolerance test (OGTT) data in 4 normal rabbits through the Pearson's correlation coefficient in each normal rabbit.

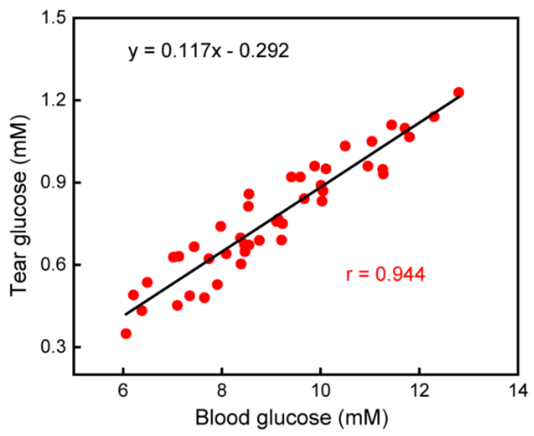

Supplementary Figure 17. Comprehensive Pearson's correlation analysis of all measured oral glucose tolerance test (OGTT) data between tear glucose and blood glucose level in 4 normal rabbits.

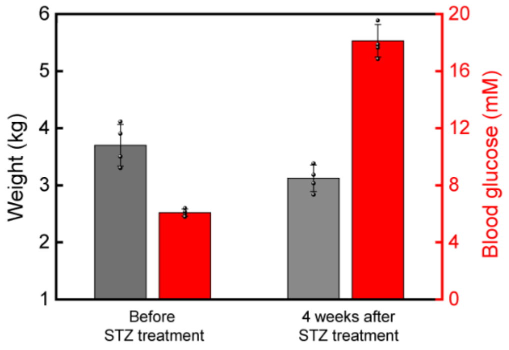

Supplementary Figure 18. Change in the average of weight and blood glucose of rabbits (n = 4) after STZ treatment. The error bars indicate mean ± standard error of the mean.

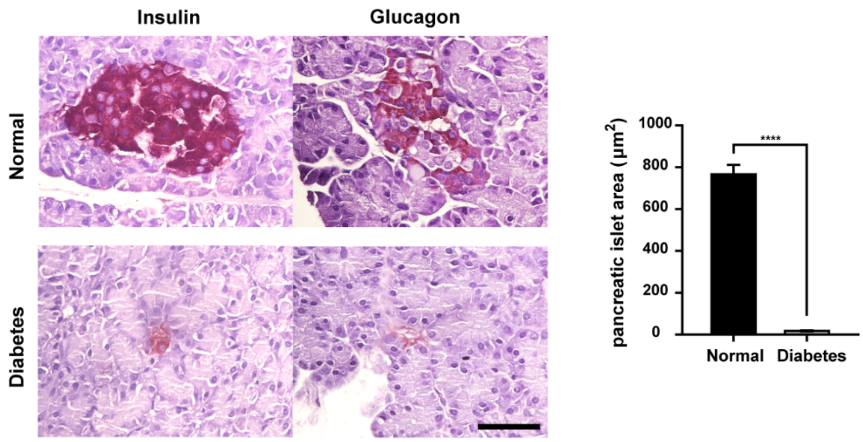

Supplementary Figure 19. Comparison of pancreatic islet between normal and diabetic rabbits by magnified images of staining of the pancreatic islet with insulin and glucagon (left) and pancreatic islet area (n = 10) (right). Scale bar, 50 μm. Significant differences were analyzed with the unpaired student's t-test and marked as **** ($p < 0.0001$). The error bars indicate mean ± standard error of the mean.

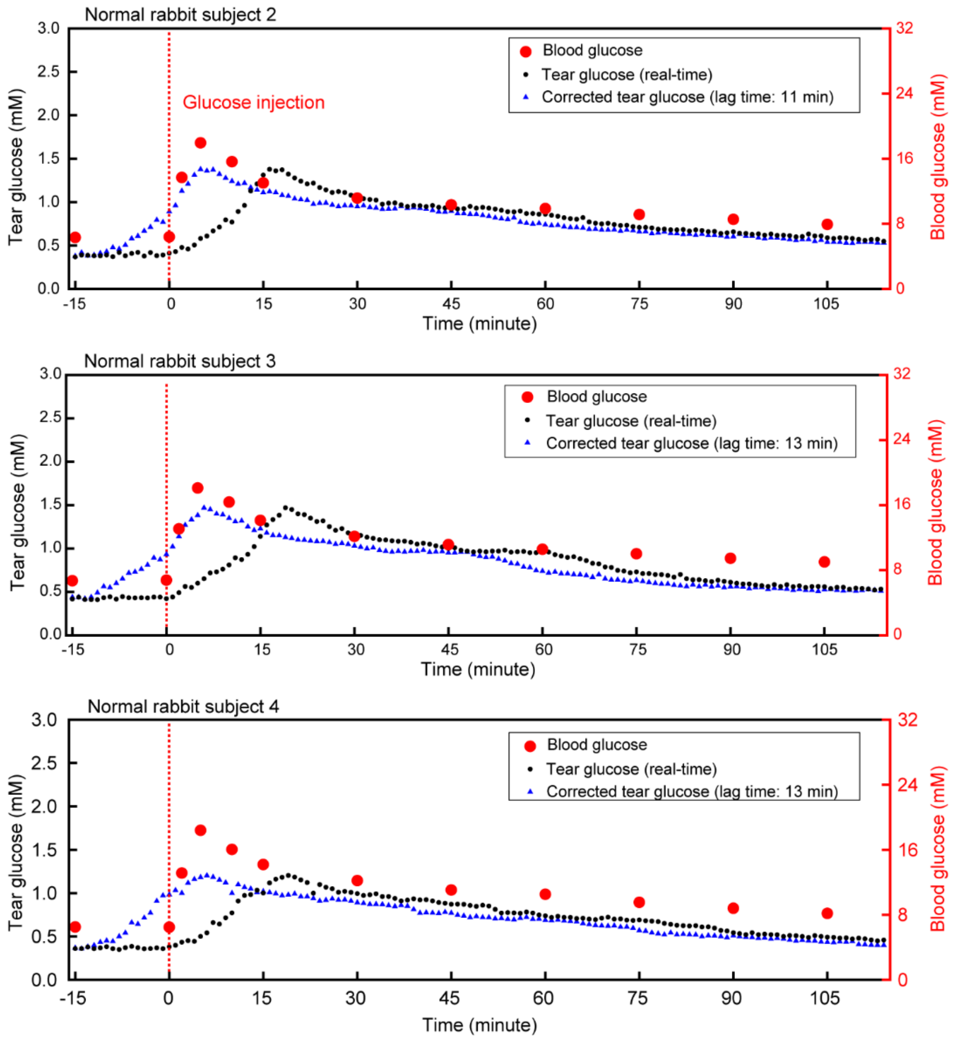

Supplementary Figure 20. Real-time measurement of tear glucose and blood glucose of each normal rabbit after intravenous injection of glucose.

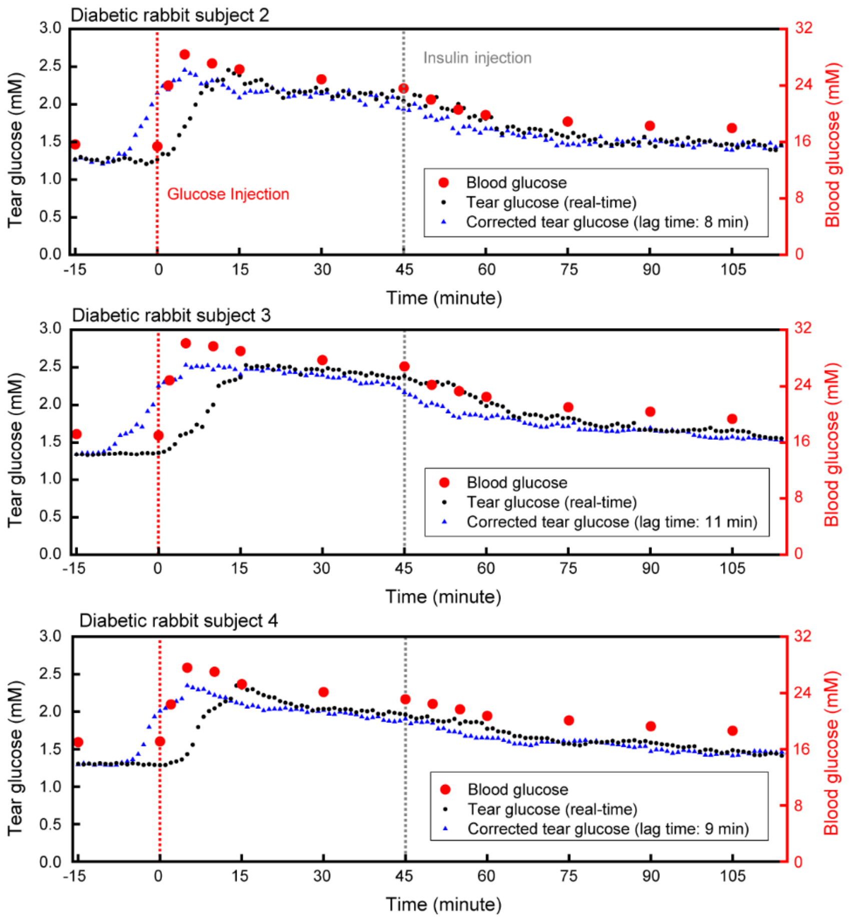

Supplementary Figure 21. Real-time measurement of tear glucose and blood glucose of each diabetic rabbit after intravenous injection of glucose and insulin.

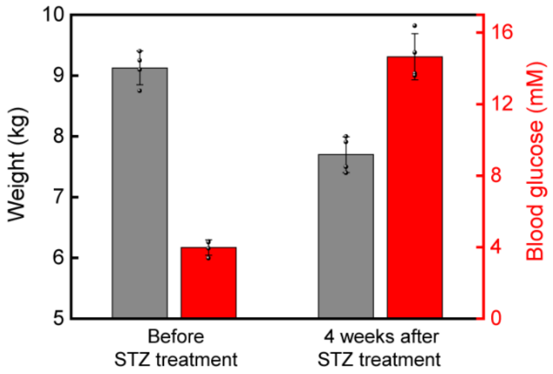

Supplementary Figure 22. Change in the average of weight and blood glucose of beagles (n = 4) after STZ treatment. The error bars indicate mean ± standard error of the mean.

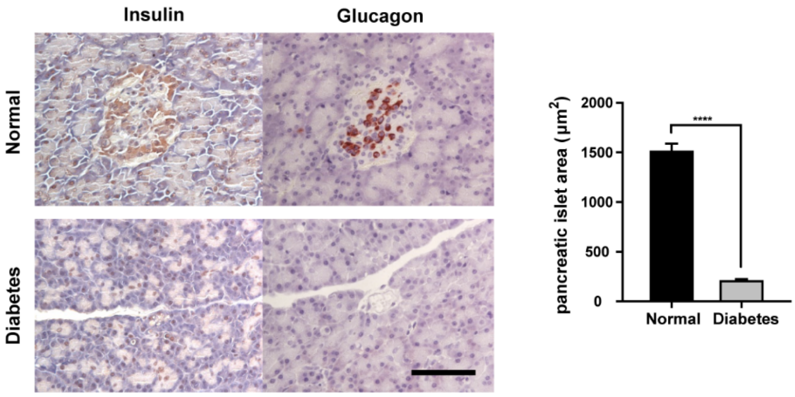

Supplementary Figure 23. Comparison of pancreatic islet between normal and diabetic beagles by magnified images of staining pancreatic islet with insulin and glucagon (left) and pancreatic islet area (n = 10) (right). Scale bar, 50 μm. Significant differences were analyzed with the unpaired student's t-test and marked as **** (p <0.0001). The error bars indicate mean ± standard error of the mean.

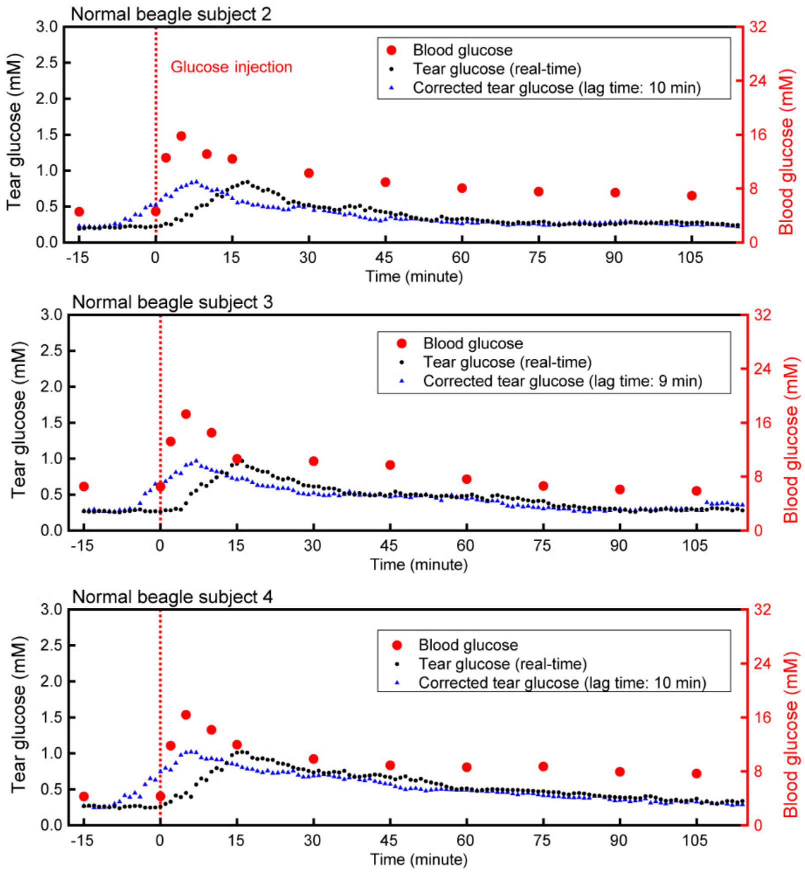

Supplementary Figure 24. Real-time measurement of tear glucose and blood glucose of each normal beagle after intravenous injection of glucose.

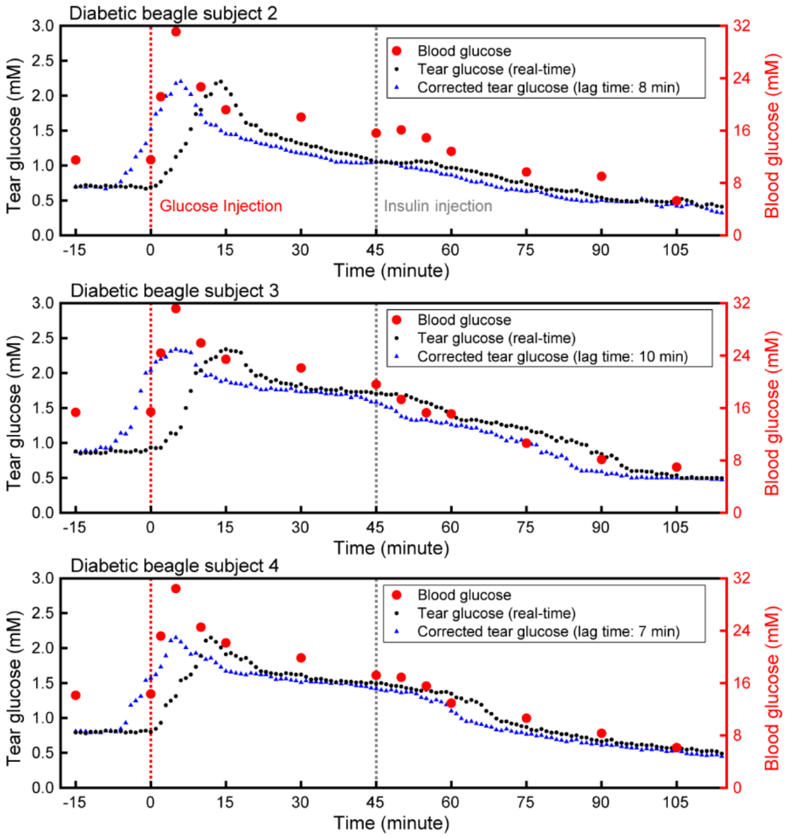

Supplementary Figure 25. Real-time measurement of tear glucose and blood glucose of each diabetic beagle after intravenous injection of glucose and insulin.

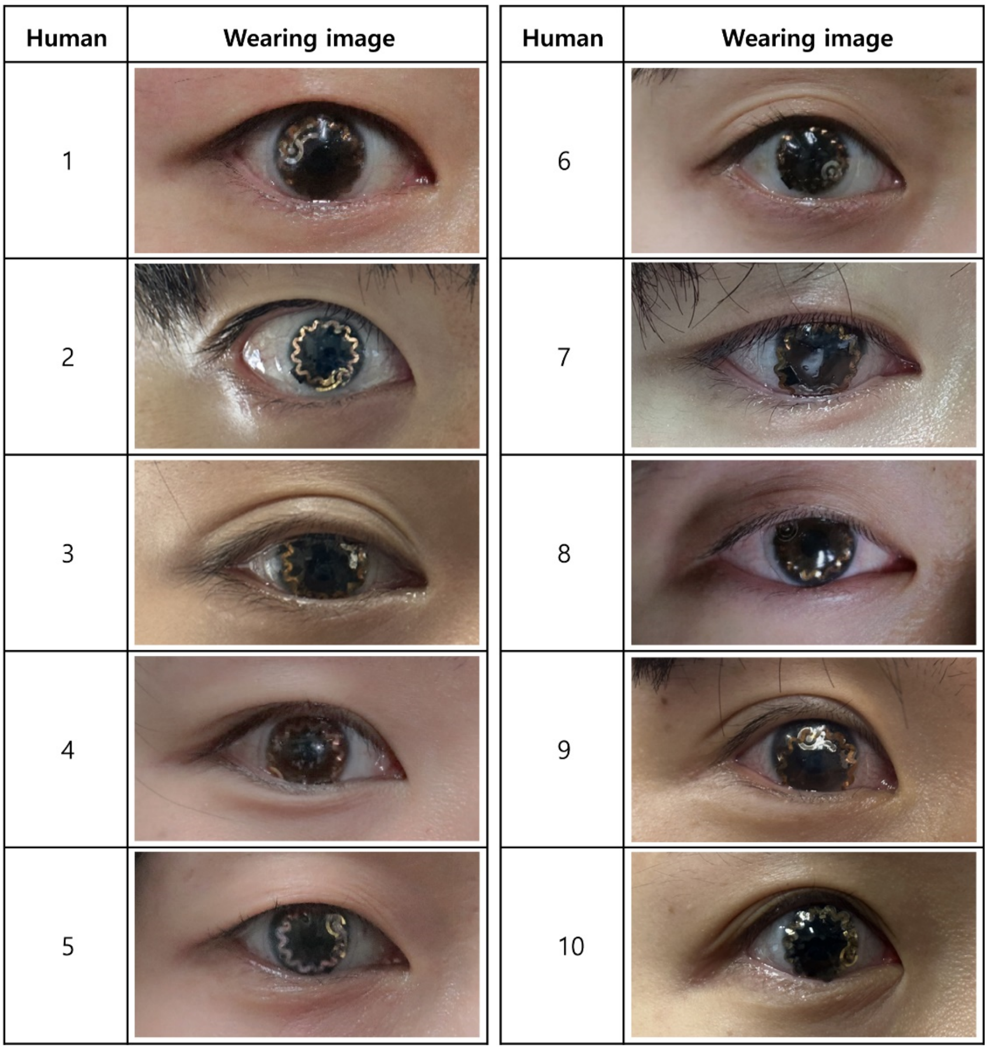

Supplementary Figure 26. Images of ten healthy participants wearing the SCL.

Human	Wearing image	Human	Wearing image
1	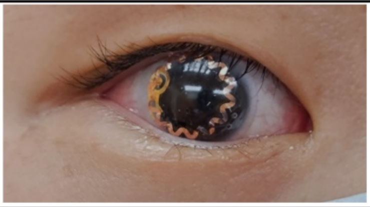	6	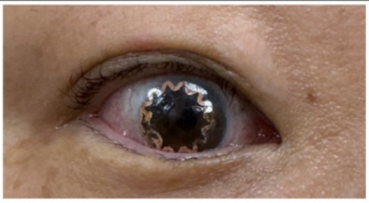
2	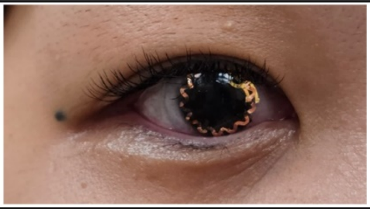	7	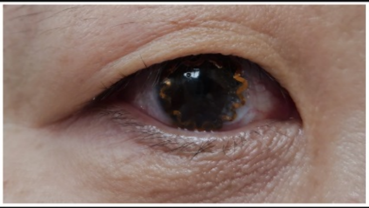
3	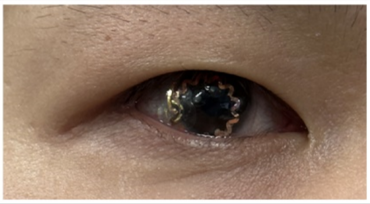	8	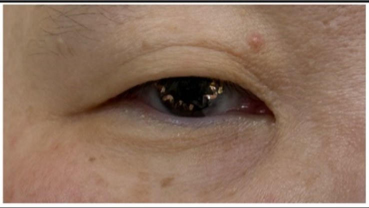
4	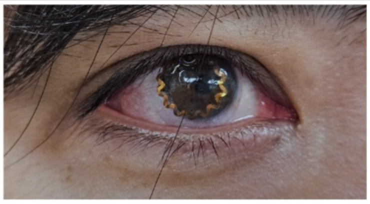	9	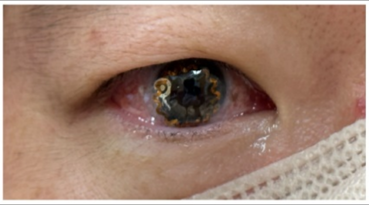
5	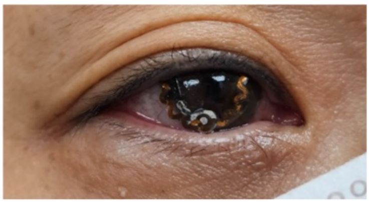	10	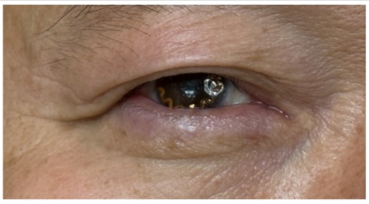

Supplementary Figure 27. Images of ten diabetic participants wearing the SCL.

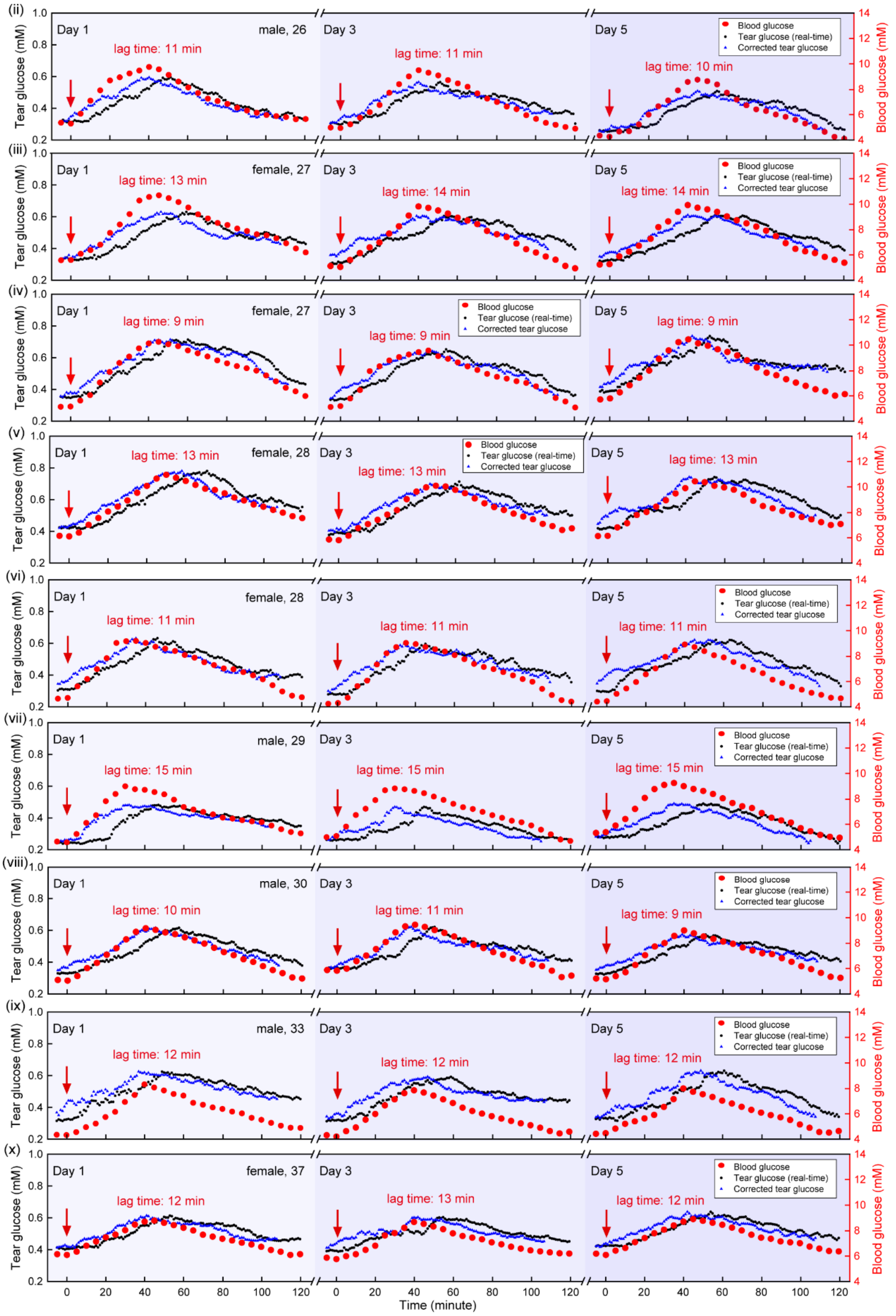

Supplementary Figure 28. Real-time data of monitoring TG and BG level in healthy participants (ii-x) after intake of a soft drink on day 1, 3, and 5.

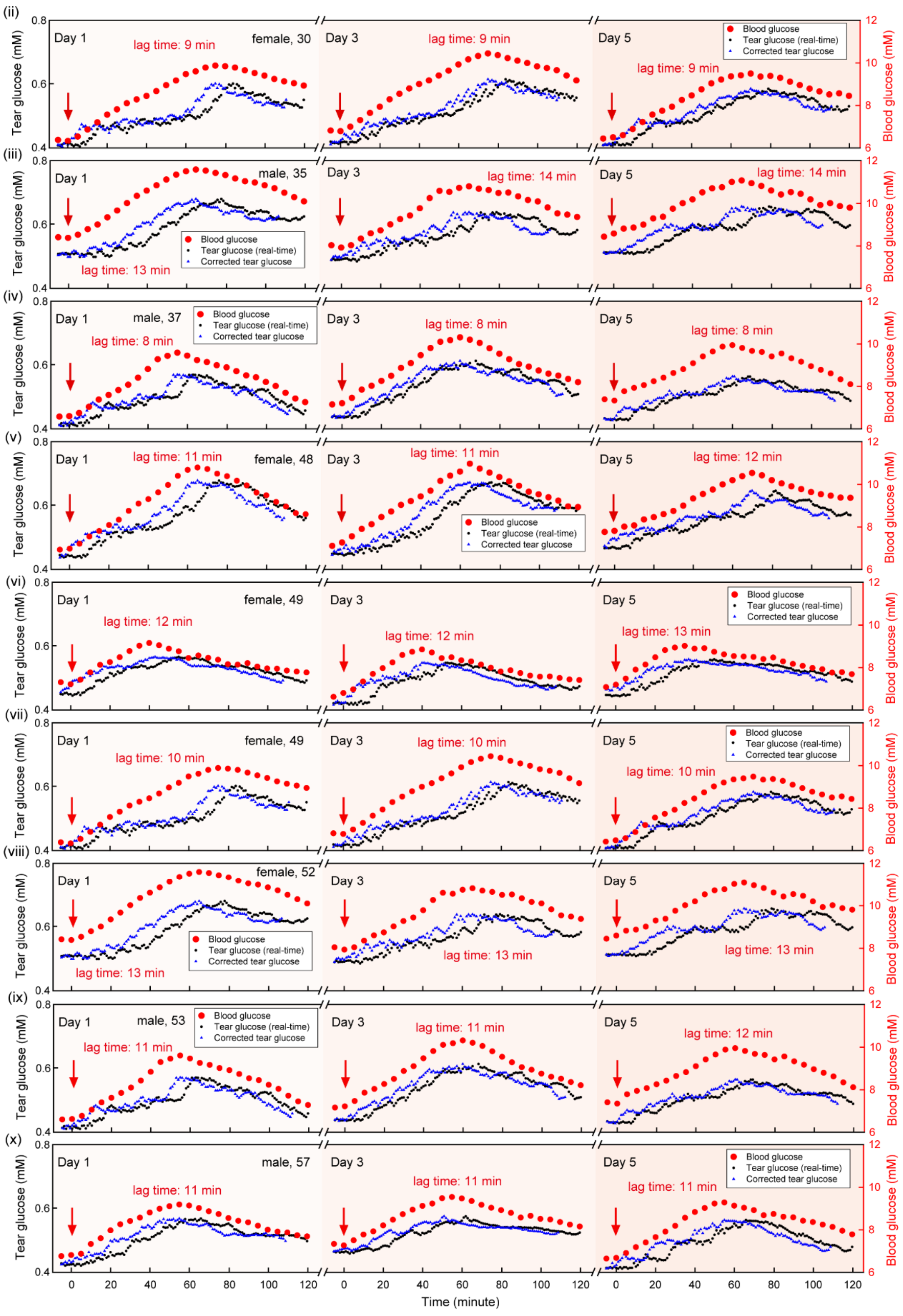

Supplementary Figure 29. Real-time data of monitoring TG and BG level in diabetic participants (ii-x) after intake of a nutrition beverage on day 1, 3, and 5.

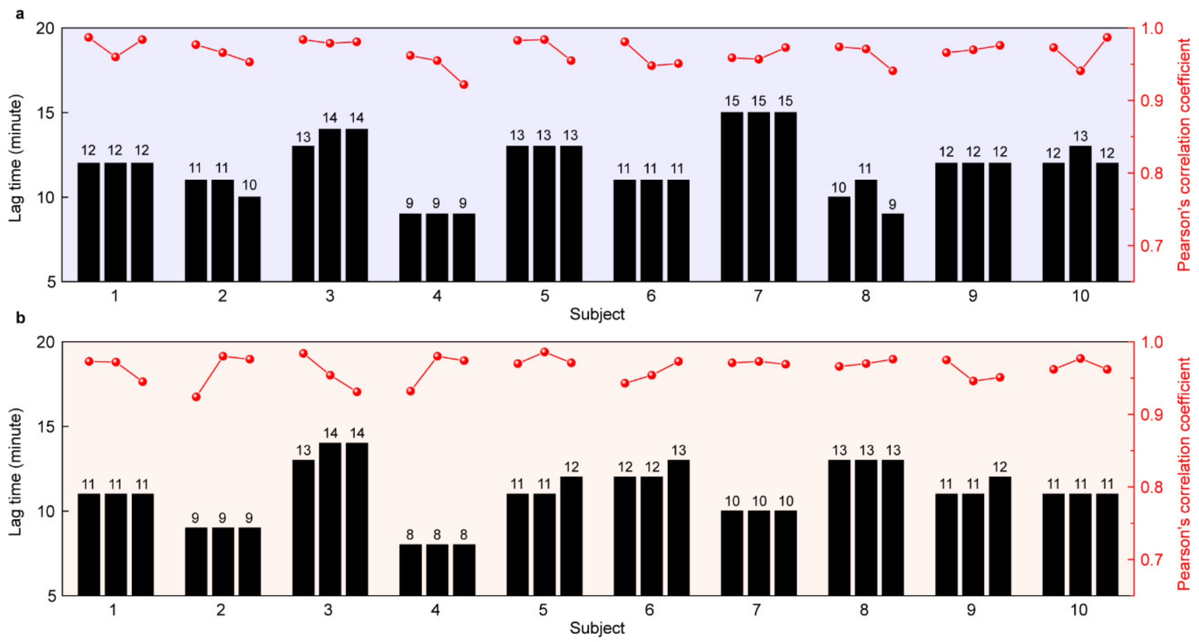

Supplementary Figure 30. The lag time and Pearson's correlation coefficient on day 1, 3, and 5. a, Healthy participants (n = 10). b, Diabetic participants (n = 10)

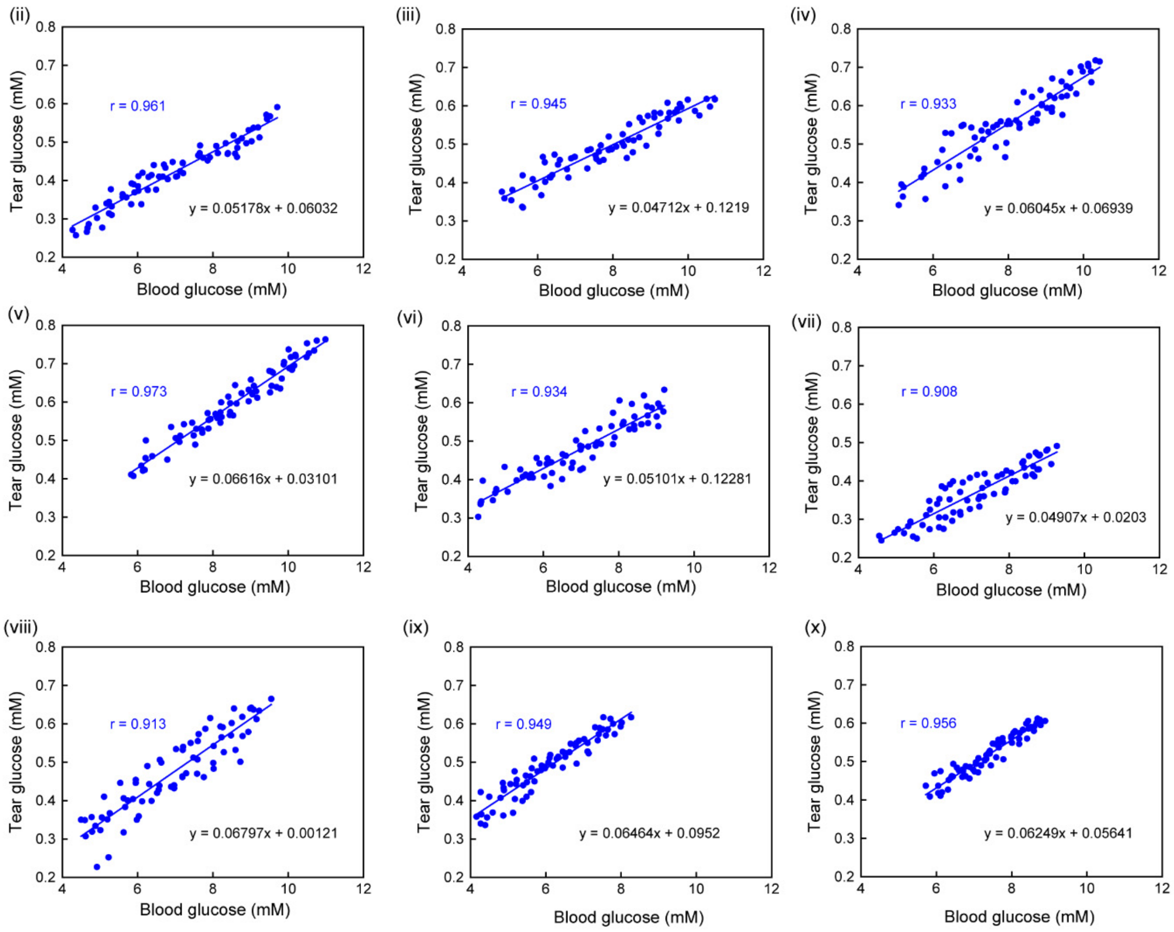

Supplementary Figure 31. Pearson's correlation analysis between TG and BG in healthy participants (ii-x), measured on day 1, 3, and 5.

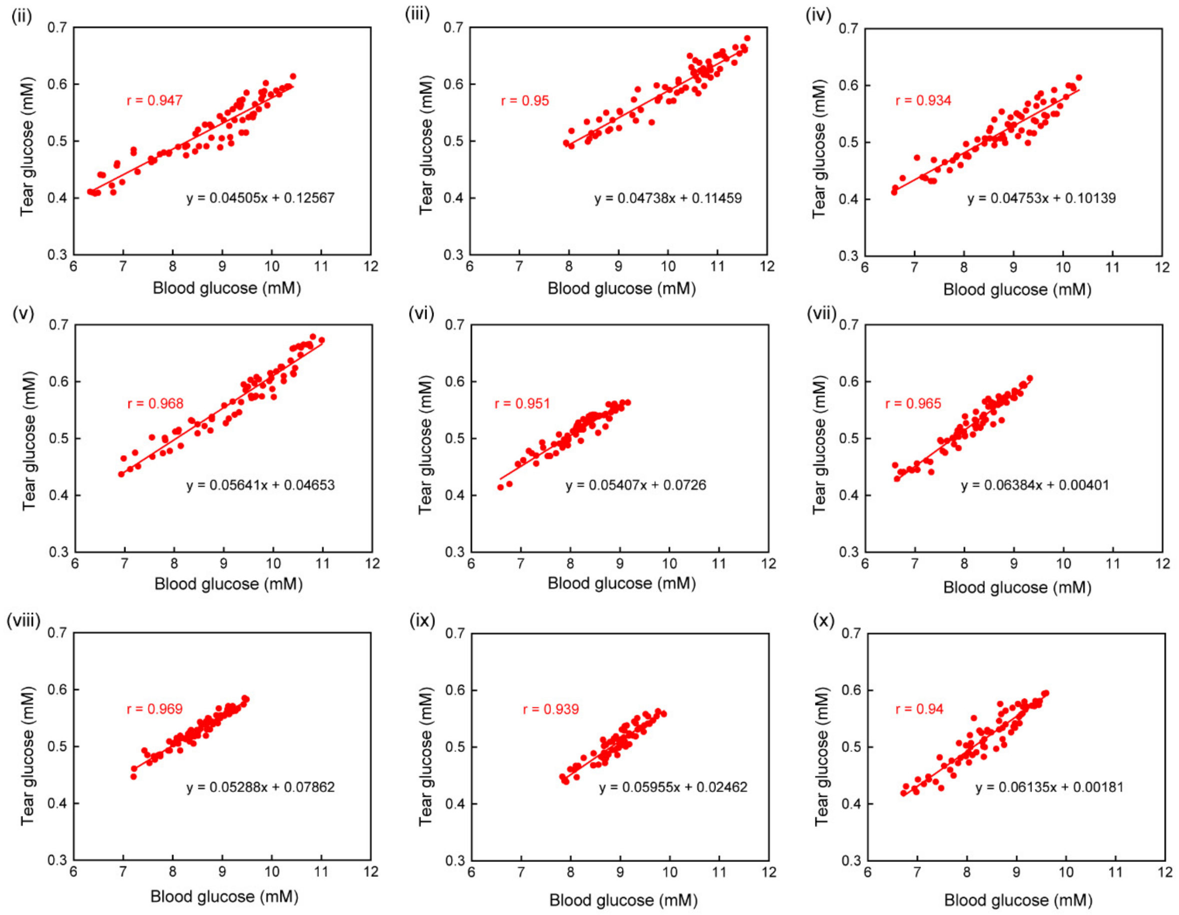

Supplementary Figure 32. Pearson's correlation analysis between TG and BG in diabetic participants (ii-x), measured on day 1, 3, and 5.

Supplementary Table 1. Comparison of various studies analyzing the correlation between tear glucose and blood glucose

Paper	Subject	Method	Measuring term (TG)	Correlation analysis	Lag time
Lewis et al.[1] (1958)	Human (diabetes)	Sticks (electrochemical method)	Single point	no correlation	Unclear
Sen et al.[2] (1980)	Human (normal, diabetes)	Microcapillary tear collection (electrochemical method)	Single point	no correlation (normal r=0.17, diabetes r=-0.03)	Unclear
Daum et al.[3] (1982)	Human (normal)	Microcapillary tear collection (electrochemical method)	1 hour term	r= 0.53	Unclear
LeBlanc et al.[4] (2005)	Human (diabetes)	Microcapillary tear collection (chromatography)	Single point	no correlation (r^2=0.052)	Unclear
Lane et al.[5] (2006)	Human (normal, diabetes)	Microcapillary tear collection (chromatography)	15 min term	normal with fasting r=0.00, normal with no fasting r=0.75, diabetic with fasting r=0.80, diabetic with no fasting r=0.88	30 min
Baca et al.[6] (2007)	Human (normal)	Microcapillary tear collection (mass spectrometry)	15 min term	qualitatively correlated	20 min
Iguchi et al.[7] (2007)	Rabbit	Wired electrochemical sensor	Continuous	qualitatively correlated	Unclear
Baca et al.[8] (2007)	Human (normal)	Microcapillary tear collection (chromatography)	Single point	r = 0.5	Unclear
Yan et al.[9] (2011)	Rabbit (normal)	Electrochemical sensor coupled with capillary	30 min term	r^2=0.4867	Unclear
Belle et al.[10] (2016)	Rabbit (normal)	Wired electrochemical sensor	20 min term	r^2=0.7544	13 min
Agustini et al.[11] (2017)	Human (normal)	Wired electrochemical sensor	Single point	r^2=0.961	Unclear
Aihara et al.[12] (2021)	Human (normal, diabetes)	Microcapillary tear collection (chromatography)	Single point	Only diabetes (p=0.054)	Unclear
Kownacka et al.[13] (2018)	Sheep (normal), Human (diabetes)	Wired electrochemical sensor	3 min term	95% in A, B region of error grid	Unclear
Sempionatto et al.[14] (2019)	Human (normal)	Eyeglasses (electrochemical sensor)	Single point	r=0.63	Unclear
Lee et al.[15] (2019)	Rabbit (normal)	Modified accu-chek	15 min term	r^2=0.764	Unclear

		(electrochemical sensor)			
Keum et al.[16] (2020)	Rabbit (normal, diabetes)	Contact lens (electrochemical sensor)	3 hours term	Qualitatively correlated	Unclear
Kim et al.[17] (2020)	Mouse (diabetes)	Contact lens (reflection spectrum)	Single point	r^2=0.76	Unclear
Geelhoed-Duijvestijn et al.[18] (2021)	Human (diabetes)	Wired electrochemical sensor	Continuous	99.7% in A, B region of error grid	15 min
Jeon et al.[19] (2021)	Mouse (normal, diabetes), Human (normal, diabetes)	Contact lens (colorimetric)	Single point	r=0.71 (mouse) r=0.72 (human)	Unclear
Lee et al.[20] (2022)	Beagle (normal)	Microcapillary tear collection (colorimetric)	5 min term	r=0.86	10 min
Kim et al.[21] (2022)	Rabbit (normal, diabetes)	Contact lens (electrochemical sensor)	5 min term	r=0.82 94.9% in A, B region of error grid	10 min
Our study	Rabbit (normal, diabetes), Beagle (normal, diabetes), Human (normal)	Contact lens (electrochemical sensor)	Continuous	r=0.946/0.944 (normal/diabetic rabbit), r=0.961/0.924 (normal/diabetic beagle), r=0.954 (normal human) All measured data in A, B region of error grid	Personalized for all subjects

Supplementary References

1. Lewis, J. G. & Stephens, P. J. TEAR GLUCOSE IN DIABETICS. *Br. J. Ophthalmol.* **42**, 754–758 (1958).
2. Sen, D. K. & Sarin, G. S. Tear glucose levels in normal people and in diabetic patients. *Br. J. Ophthalmol.* **64**, 693–695 (1980).
3. Daum, K. M. & Hill, R. M. Human tear glucose. *Invest. Ophthalmol. Vis. Sci.* **22**, 509–514 (1982).
4. LeBlanc, J. M., Haas, C. E., Vicente, G. & Colon, L. A. Evaluation of lacrimal fluid as an alternative for monitoring glucose in critically ill patients. *Intensive Care Med.* **31**, 1442–1445 (2005).
5. Lane, J. D., Krumholz, D. M., Sack, R. A. & Morris, C. Tear Glucose Dynamics in Diabetes Mellitus. *Curr. Eye Res.* **31**, 895–901 (2006).
6. Baca, J. T., Finegold, D. N. & Asher, S. A. Tear Glucose Analysis for the Noninvasive Detection and Monitoring of Diabetes Mellitus. *Ocul. Surf.* **5**, 280–293 (2007).
7. Iguchi, S. *et al.* A flexible and wearable biosensor for tear glucose measurement. *Biomed. Microdevices* **9**, 603–609 (2007).
8. Baca, J. T. *et al.* Mass Spectral Determination of Fasting Tear Glucose Concentrations in Nondiabetic Volunteers. *Clin. Chem.* **53**, 1370–1372 (2007).
9. Yan, Q. *et al.* Measurement of Tear Glucose Levels with Amperometric Glucose Biosensor/Capillary Tube Configuration. *Anal. Chem.* **83**, 8341–8346 (2011).
10. Belle, J. T. L. *et al.* Self-monitoring of tear glucose: the development of a tear based glucose sensor as an alternative to self-monitoring of blood glucose. *Chem. Commun.* **52**, 9197–9204 (2016).

11. Agustini, D., Bergamini, M. F. & Marcolino-Junior, L. H. Tear glucose detection combining microfluidic thread based device, amperometric biosensor and microflow injection analysis. *Biosens. Bioelectron.* **98**, 161–167 (2017).
12. Aihara, M. *et al.* Association between tear and blood glucose concentrations: Random intercept model adjusted with confounders in tear samples negative for occult blood. *J. Diabetes Investig.* **12**, 266–276 (2021).
13. Kownacka, A. E. *et al.* Clinical Evidence for Use of a Noninvasive Biosensor for Tear Glucose as an Alternative to Painful Finger-Prick for Diabetes Management Utilizing a Biopolymer Coating. *Biomacromolecules* **19**, 4504–4511 (2018).
14. Sempionatto, J. R. *et al.* Eyeglasses-based tear biosensing system: Non-invasive detection of alcohol, vitamins and glucose. *Biosens. Bioelectron.* **137**, 161–170 (2019).
15. Lee, S. H., Cho, Y. C. & Bin Choy, Y. Noninvasive Self-diagnostic Device for Tear Collection and Glucose Measurement. *Sci. Rep.* **9**, 4747 (2019).
16. Keum, D. H. *et al.* Wireless smart contact lens for diabetic diagnosis and therapy. *Sci. Adv.* **6**, eaba3252 (2020).
17. Kim, S., Jeon, H.-J., Park, S., Lee, D. Y. & Chung, E. Tear Glucose Measurement by Reflectance Spectrum of a Nanoparticle Embedded Contact Lens. *Sci. Rep.* **10**, 8254 (2020).
18. Geelhoed-Duijvestijn, P. *et al.* Performance of the Prototype NovioSense Noninvasive Biosensor for Tear Glucose in Type 1 Diabetes. *J. Diabetes Sci. Technol.* **15**, 1320–1325 (2021).
19. Jeon, H.-J. *et al.* Optical Assessment of Tear Glucose by Smart Biosensor Based on Nanoparticle Embedded Contact Lens. *Nano Lett.* **21**, 8933–8940 (2021).
20. Lee, E. *et al.* Quantification of tear glucose levels and their correlation with blood glucose levels in dogs. *Vet. Med. Sci.* **8**, 1816–1824 (2022).

21. Kim, S.-K. *et al.* Bimetallic Nanocatalysts Immobilized in Nanoporous Hydrogels for Long-Term Robust Continuous Glucose Monitoring of Smart Contact Lens. *Adv. Mater.* **34**, 2110536 (2022).
