# Supplementary material for: In-depth correlation analysis between tear glucose and blood glucose using a wireless smart contact lens
Source: Nat Commun. 2024 Apr 2;15:2828. doi: 10.1038/s41467-024-47123-9 (PMC10987615; doi:10.1038/s41467-024-47123-9)
Supplement: Supplementary file 3 — Description of Additional Supplementary Files [file 41467_2024_47123_MOESM3_ESM.pdf]

Description of Additional Supplementary Files

Supplementary Movie 1. In-vivo test using a live normal rabbit for the simultaneous monitoring of tear glucose and blood glucose.

Supplementary Movie 2. In-vivo test using a live normal beagle for the simultaneous monitoring of tear glucose and blood glucose.

Supplementary Movie 3. Human pilot trial of smart contact lens for simultaneous monitoring of tear glucose and blood glucose. Here, the participant’s face was blocked using a black bar, except for eyes, to infringe the portrait right.
