# Supplementary material for: In-depth correlation analysis between tear glucose and blood glucose using a wireless smart contact lens
Source: Nat Commun. 2024 Apr 2;15:2828. doi: 10.1038/s41467-024-47123-9 (PMC10987615; doi:10.1038/s41467-024-47123-9)
Supplement: Supplementary file 7 — Reporting Summary [file 41467_2024_47123_MOESM7_ESM.pdf]

nature portfolio

Corresponding author(s): Jang-Ung Park

Last updated by author(s): Jan 30, 2024

Reporting Summary

Nature Portfolio wishes to improve the reproducibility of the work that we publish. This form provides structure for consistency and transparency in reporting. For further information on Nature Portfolio policies, see our Editorial Policies and the Editorial Policy Checklist.

Statistics

For all statistical analyses, confirm that the following items are present in the figure legend, table legend, main text, or Methods section.

n/a	Confirmed	
☐	☒	The exact sample size (*n*) for each experimental group/condition, given as a discrete number and unit of measurement
☐	☒	A statement on whether measurements were taken from distinct samples or whether the same sample was measured repeatedly
☒	☐	The statistical test(s) used AND whether they are one- or two-sided<br>*Only common tests should be described solely by name; describe more complex techniques in the Methods section.*
☒	☐	A description of all covariates tested
☒	☐	A description of any assumptions or corrections, such as tests of normality and adjustment for multiple comparisons
☐	☒	A full description of the statistical parameters including central tendency (e.g. means) or other basic estimates (e.g. regression coefficient) AND variation (e.g. standard deviation) or associated estimates of uncertainty (e.g. confidence intervals)
☐	☒	For null hypothesis testing, the test statistic (e.g. *F*, *t*, *r*) with confidence intervals, effect sizes, degrees of freedom and *P* value noted<br>*Give P values as exact values whenever suitable.*
☒	☐	For Bayesian analysis, information on the choice of priors and Markov chain Monte Carlo settings
☒	☐	For hierarchical and complex designs, identification of the appropriate level for tests and full reporting of outcomes
☐	☒	Estimates of effect sizes (e.g. Cohen's *d*, Pearson's *r*), indicating how they were calculated

Our web collection on statistics for biologists contains articles on many of the points above.

Software and code

Policy information about availability of computer code

Data collection	VersaStudio<br>Android studio
Data analysis	Origin 2022b<br>ImageJ<br>Matlab R2020a<br>Prism version 8<br>Excel<br>G* power software

For manuscripts utilizing custom algorithms or software that are central to the research but not yet described in published literature, software must be made available to editors and reviewers. We strongly encourage code deposition in a community repository (e.g. GitHub). See the Nature Portfolio guidelines for submitting code & software for further information.

Data

Policy information about availability of data

All manuscripts must include a data availability statement. This statement should provide the following information, where applicable:

- Accession codes, unique identifiers, or web links for publicly available datasets
- A description of any restrictions on data availability
- For clinical datasets or third party data, please ensure that the statement adheres to our policy

The main data supporting the findings of this study are available within the paper and its Supplementary Information. Any additional requests for information can be directed to, and will be fulfilled by, the corresponding authors. Source data are provided with this paper. Source data are available at Figshare (https://doi.org/10.6084/m9.figshare.25288405). Source data are provided with this paper for reproducing all Figures in the manuscript and Supplementary Information.

Research involving human participants, their data, or biological material

Policy information about studies with human participants or human data. See also policy information about sex, gender (identity/presentation), and sexual orientation and race, ethnicity and racism.

Reporting on sex and gender	Sex and gender were not considered in the study design, and the recruited population characteristics were close to evenly split across males and females. There are no self-selection biases or other biases on sex or gender.
Reporting on race, ethnicity, or other socially relevant groupings	There are no any relevant grouping experiments for race, ethnicity, or other socially.
Population characteristics	All participants are over 18 years old. The ten healthy participants (five male and five female) and ten diabetic participants (five male and five female) were recruited.
Recruitment	The participants (healthy and diabetic) were recruited from campus of Yonsei University and neighboring communities through advertisement by posted notices, word of mouth. There were no self-selection biased or other biases.
Ethics oversight	All human studies were approved by the Institutional Review Board (IRB) of Yonsei University and Kyungpook National University Hospital approved the human pilot study protocol (7001988-202311-HR-1451-06, KNUH 2022-11-003-005) which was conducted according to the Declaration of Helsinki, and all experimental procedures were performed with the informed consent of the participants.

Note that full information on the approval of the study protocol must also be provided in the manuscript.

Field-specific reporting

Please select the one below that is the best fit for your research. If you are not sure, read the appropriate sections before making your selection.

☒ Life sciences ☐ Behavioural & social sciences ☐ Ecological, evolutionary & environmental sciences

For a reference copy of the document with all sections, see nature.com/documents/nr-reporting-summary-flat.pdf

Life sciences study design

All studies must disclose on these points even when the disclosure is negative.

Sample size

In the pilot test of rabbits, when comparing the maximum blood glucose levels between the control and diabetes groups during IVGTT, the control group exhibited 156.67 +/- 7.53 mg/dl, while the diabetes group showed 535.67 +/- 34.51 mg/dl. Based on these results, the G*Power program (Ver. 3.1) determined a minimum sample size of 2 for each group using a two-tailed Student's t-test, with a significance level of 5% and a statistical power exceeding 95%.
Similarly, analysis of islet area through insulin immunostaining revealed a significant difference, with the control group measuring 780.20 +/- 162.27 µm^2 and the diabetes group measuring 17.32 +/- 8.54 µm^2. Again, using a significance level of 5% and a statistical power exceeding 95%, the minimum sample size required for the experiment was determined to be 3.
Considering the risk of fatalities associated with diabetes induction experiments in rabbits, an additional 20% of animals were added, resulting in a total of 4 animals per group for the experiment. None of the 4 animals in each group experienced fatalities, and all 4 animals were used to derive the results.
Similarly, in the beagle experiments, the IVGTT results showed blood glucose levels of 16.54 +/- 0.32 mM in the control group and 30.465 +/- 0.75 mM in the diabetes group. Islet area analysis through insulin immunostaining revealed areas of 1506.51 +/- 271.80 µm^2 in the control group and 203.17 +/- 70.12 µm^2 in the diabetes group. The minimum sample size for each group was determined to be 2 and 3, respectively, using the same method. Ultimately, 4 animals per group were chosen for the experiment, with no fatalities observed.
Furthermore, similar conclusions were drawn from previous experiments, as evidenced by our previously submitted manuscripts.
-Reference-
Faul, F., Erdfelder, E., Lang, A.-G., & Buchner, A. (2007). G*Power 3: A flexible statistical power analysis program for the social, behavioral, and biomedical sciences. Behavior Research Methods, 39, 175-191.
Faul, F., Erdfelder, E., Buchner, A., & Lang, A.-G. (2009). Statistical power analyses using G*Power 3.1: Tests for correlation and regression analyses. Behavior Research Methods, 41, 1149-1160.

H. Song, H. Shin, H. Seo, W. Park, B. J. Joo, J. Kim, J. Kim, H. K. Kim, J. Kim, J.-U. Park, Wireless Non-Invasive Monitoring of Cholesterol Using a Smart Contact Lens. Adv. Sci. 2022, 9, 2203597.

Data exclusions	No data were excluded from the analyses.
Replication	At least three biologically independent experiments were performed in each case of this study, and all experimental findings were reliably reproduced at least three times.
Randomization	The experiment was conducted by randomly assigning four experimental animals (rabbits or beagles) to a control group and a diabetes group.
Blinding	Blinding was not relevant, because a blinding process wouldn't influence the sampling result.

Reporting for specific materials, systems and methods

We require information from authors about some types of materials, experimental systems and methods used in many studies. Here, indicate whether each material, system or method listed is relevant to your study. If you are not sure if a list item applies to your research, read the appropriate section before selecting a response.

Materials & experimental systems

n/a	Involved in the study	
☐	☒	Antibodies
☐	☒	Eukaryotic cell lines
☒	☐	Palaeontology and archaeology
☐	☒	Animals and other organisms
☒	☐	Clinical data
☒	☐	Dual use research of concern
☒	☐	Plants

Methods

n/a	Involved in the study	
☒	☐	ChIP-seq
☒	☐	Flow cytometry
☒	☐	MRI-based neuroimaging

Antibodies

Antibodies used	Monoclonal Anti-Insulin antibody produced in mouse IgG1 (Sigma-Aldrich, USA Cat# I2018-2ML, Source #0000091067, Batch # 0000121541, Clone K36AC10, monoclonal), Dilution 1:200<br>rabbit anti-glucagon polyclonal antibody (MyBioSource, USA, Cat# MBS5314403, Lot# X22011012), Dilution 1:200<br>mouse anti-rabbit IgG-HRP (Santa Cruz Biotechnology, USA, Cat# sc-2357, Lot# F2921) Dilution 1:400<br>m-IgGκ BP-HRP (Santa Cruz Biotechnology, USA, Cat# sc-516102, Lot # G0621) Dilution 1:400<br>All antibody products were purchased and used in experiments.
Validation	Monoclonal Anti-Insulin antibody produced in mouse: Dot blot, immunohistochemistry, radioimmunoassay<br>rabbit anti-glucagon polyclonal antibody : Western Blot, Immunohistochemistry, Immunofluorescence, Immunohistochemistry<br>mouse anti-rabbit IgG-HRP : Western Blot, Immunohistochemistry<br>m-IgGκ BP-HRP: Western Blot, Immunohistochemistry<br><br>Validation statement on the manufacturer's website:<br>The primary Insulin antibody used in the manuscript was validated based on the supplier statement (https://www.sigmaaldrich.com/KR/ko/product/sigma/i2018)<br>The primary glucagon antibody used in the manuscript was validated based on the supplier statement (https://www.mybiosource.com/polyclonal-antibody/glucagon/5314403)<br><br>Secondary antibodies<br>mouse anti-rabbit IgG-HRP (https://datasheets.scbt.com/sc-2357.pdf)<br>m-IgGκ BP-HRP (https://datasheets.scbt.com/sc-516102.pdf)

Eukaryotic cell lines

Policy information about cell lines and Sex and Gender in Research

Cell line source(s)	The human corneal epithelial cell line (HCE-2, #CRL-11135, clone 50.B1, ATCC) and human conjunctival epithelial cell line (HCECs, #CCL- 20-2, clone 1-5c-4, ATCC) were obtained from the American Type Culture Collection (ATCC, Manassas, VA, USA)
Authentication	In this study, the cell lines used did not undergo ATCC's STR testing, however, we made a concerted effort to choose cell lines with the lowest possible passage numbers (passage 2-3) after purchase. Subsequently, we cultured and passaged the cells according to the manufacturer's recommended protocol.
Mycoplasma contamination	Regular mycoplasma contamination testing was conducted, and the cell lines used were free from mycoplasma contamination.

Commonly misidentified lines (See ICLAC register)	No commonly misidentified cell lines were used.

Animals and other research organisms

Policy information about studies involving animals; ARRIVE guidelines recommended for reporting animal research, and Sex and Gender in Research

Laboratory animals	Male New Zealand white rabbits (3 kg, 4 months, Specific pathogen Free grade, four normal rabbits and four diabetic rabbits) and male beagle dogs (9 kg, 9 months, Conventional grade, four normal beagles and four diabetic beagles) were used. Beagle dogs were purchased from ORIENT BIO Inc(Korea, Republic of) and New Zealand white rabbits were purchased from SAMTACO BIO KOREA (Korea, Republic of).<br>All experimental animals were housed at the K-MEDIHUB Preclinical Research Center located in Daegu, South Korea. The animal facility maintained controlled environmental conditions with relative humidity of 50% ± 5%, temperature of 23°C ± 2.5°C, and a 12-h light/dark cycle. Animals were housed in stainless steel cages equipped with automatic floor cleaning. During the breeding period, standard feed and clean water suitable for each species were provided freely.<br><br>Laboratory animal vendor's website;<br>ORIENT BIO Inc.(http://www.orientbio.co.kr:48080/eng/)<br>SAMTACO BIO KOREA (http://www.samtako.com/)
Wild animals	This study did not involve wild animals.
Reporting on sex	Sex was not considered in the study.
Field-collected samples	This study did not involve samples collected fron the field.
Ethics oversight	All in-vivo tests using rabbits and beagles were conducted according to the guidelines of the Institute of Animal Care and Use Committee of Yonsei University (IACUC-202106-1276-04) and Daegu-Gyeongbuk Medical Innovation Foundation (DGMIF) (DGMIF-20071503-03, KMEDI-22012701-00).

Note that full information on the approval of the study protocol must also be provided in the manuscript.

Plants

Seed stocks	The study did not involve plant samples.
Novel plant genotypes	The study did not involve plant samples.
Authentication	The study did not involve plant samples.
